# Exposure to Environmental Arsenic and Emerging Risk of Alzheimer’s Disease: Perspective Mechanisms, Management Strategy, and Future Directions

**DOI:** 10.3390/toxics9080188

**Published:** 2021-08-14

**Authors:** Md. Ataur Rahman, Md. Abdul Hannan, Md Jamal Uddin, Md Saidur Rahman, Md Mamunur Rashid, Bonglee Kim

**Affiliations:** 1 Department of Pathology, College of Korean Medicine, Kyung Hee University, 1-5 Hoegidong Dongdaemungu, Seoul 02447, Korea; 2 Korean Medicine-Based Drug Repositioning Cancer Research Center, College of Korean Medicine, Kyung Hee University, Seoul 02447, Korea; 3 Global Biotechnology & Biomedical Research Network (GBBRN), Department of Biotechnology and Genetic Engineering, Faculty of Biological Sciences, Islamic University, Kushtia 7003, Bangladesh; 4 ABEx Bio-Research Center, East Azampur, Dhaka 1230, Bangladesh; hasan800920@gmail.com; 5 Department of Biochemistry and Molecular Biology, Bangladesh Agricultural University, Mymensingh 2202, Bangladesh; hannanbmb@bau.edu.bd; 6 Graduate School of Pharmaceutical Sciences, College of Pharmacy, Ewha Womans University, Seoul 03760, Korea; 7 Department of Animal Science & Technology and BET Research Institute, Chung-Ang University, Anseong 17546, Korea; shohagvet@gmail.com; 8 Department of Clinical Pharmacology and Therapeutics, Seoul National University College of Medicine and Hospital, Seoul 03080, Korea; mamun@snu.ac.kr

**Keywords:** arsenic, Alzheimer’s disease (AD), environmental risk factor, mitochondrial dysfunction, proteostasis, apoptosis, phytochemicals

## Abstract

Alzheimer’s disease (AD) is one of the most prevailing neurodegenerative diseases, characterized by memory dysfunction and the presence of hyperphosphorylated tau and amyloid β (Aβ) aggregates in multiple brain regions, including the hippocampus and cortex. The exact etiology of AD has not yet been confirmed. However, epidemiological reports suggest that populations who were exposed to environmental hazards are more likely to develop AD than those who were not. Arsenic (As) is a naturally occurring environmental risk factor abundant in the Earth’s crust, and human exposure to As predominantly occurs through drinking water. Convincing evidence suggests that As causes neurotoxicity and impairs memory and cognition, although the hypothesis and molecular mechanism of As-associated pathobiology in AD are not yet clear. However, exposure to As and its metabolites leads to various pathogenic events such as oxidative stress, inflammation, mitochondrial dysfunctions, ER stress, apoptosis, impaired protein homeostasis, and abnormal calcium signaling. Evidence has indicated that As exposure induces alterations that coincide with most of the biochemical, pathological, and clinical developments of AD. Here, we overview existing literature to gain insights into the plausible mechanisms that underlie As-induced neurotoxicity and the subsequent neurological deficits in AD. Prospective strategies for the prevention and management of arsenic exposure and neurotoxicity have also been discussed.

## 1. Introduction

**Figure 1:**
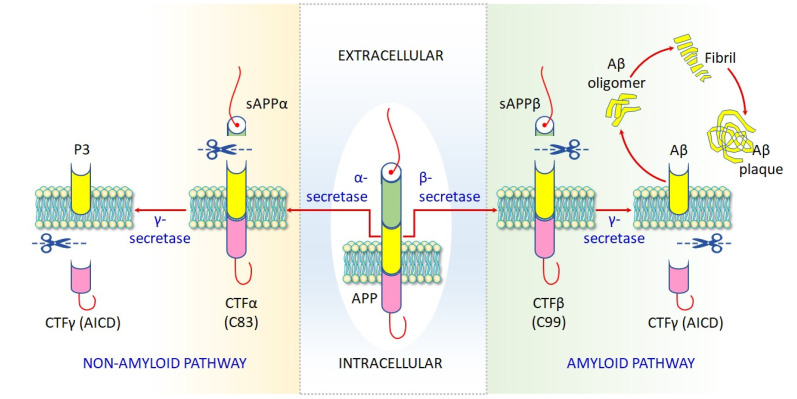
Mechanism of AD pathogenesis. The amyloid pathway is important to accumulate neurotoxic Aβ plaque via releases of β-secretase, which produces extracellular sAPPβ and C99. Cleavage of the C99 fragment by γ-secretase releases Aβ oligomer, which subsequently produces Aβ peptide. By the action of α-secretase, two fragments of sAPPα and C83 are produced by the non-amyloid pathway. The non-amyloid pathway cleavages APP via α-secretase to produce two fragments: C83, an 83 amino acid intracellular C-terminal fragment, and extracellular sAPPα, soluble amyloid precursor protein α. Cleavage of the C83 fragment by γ-secretase yields a P3 peptide and CTFγ.

Alzheimer’s disease (AD) is the most prevalent neurodegenerative disease and the prime cause of dementia among the elderly. The main pathological features are intracellular aggregates comprising phosphorylated tau protein, which forms neurofibrillary tangles (NFTs), as well as extracellular deposition of amyloid-β (Aβ) leading to the formation of senile plaques (Figure 1) [1,2,3,4]. About 70% of AD risk has been considered to be inherited, and several genes are commonly involved, while the actual causes along with molecular mechanisms have not yet been well understood [4,5]. It has been found that brains are affected by AD mostly via activated microglia, reactive astrocytes, oxidative damage, and altered proteostasis [6]. Recent observations suggest that exposure to several environmental factors may enhance the prospective risk of AD [3], although the authentic etiology of AD is not yet clear. Several investigations have emphasized that environmental risk factors may play a significant role in accelerating or decelerating the progression of AD. Such environmental risk factors include particulate air, pesticides, metal-containing nanoparticles, and numerous metalloids such as arsenic, aluminum, lead, cadmium, and mercury [3,7,8]. Arsenic has been found to be the most toxic metalloid responsible for the neurotoxicity associated with AD development in the brain, impairing cognitive function [7], although the role of As exposure in AD development has been poorly elucidated.

According to epidemiological investigations, populations around the world are increasingly recognizing that childhood is the most vulnerable stage during which a low level of As exposure has a detrimental effect on AD development [8]. Exposure has been found to be connected to learning deficiency, variations of neurotransmitter release, and behavioral deficits [9]. Inorganic arsenic (iAs) exposure through drinking water leads to the bioenergetic impairment of 3xTgAD, in an AD model, compared to counterparts, suggesting that As exposure exacerbates AD pathophysiological progression [10]. Additionally, inorganic and organic As exposures increase APP and sAPPβ expression in cholinergic SN56.B5.G4 cells [9]. Moreover, prolonged arsenic exposure causes an increase in tau phosphorylation and insoluble tau aggregates [11]. These observations suggest that As might have the capacity to enhance Aβ accumulation and tau phosphorylation to initiate senile plaque formation, leading to increased susceptibility to AD. Therefore, an exhaustive literature review has been conducted to better understand the relationship between As toxicity and AD development along with the pathomechanism in redefining the health risks of As.

## 2. Environmental Sources of Arsenic Exposure

The most abundant inorganic arsenic found in the air is arsenic trioxide (As_2_O_3_); however, it may also be found in water, soil, or food, with the most prevalent being inorganic arsenic (AsO_3_) or arsenites (AsO_2_) [12]. Gallium arsenide (GaAs) is an inorganic arsenic compound that has serious human health effects because of its widespread use in microelectronics [13]. Arsenic and other metals generally are the major source, with seafood, grains, mushrooms, and poultry as the predominant dietary types. Although seafood contains more arsenic than other meals, it is mainly called arsenobetaine in an organic form, far less dangerous than other types. The most common causes of arsenic poisoning include occupational exposure, contaminated wine or moonshine, or malicious delivery [14]. Recently, traditional Chinese herbal arsenic-enhanced remedies have been found to represent a considerable risk to health [15]. In cosmetic color, pigments used to make eye shadows, toxic substances such as arsenic are often discovered. Eyelash skin is particularly sensitive, and eczema can be caused by applying eye-shadows [16]. Arsenic particles that are water-soluble can be absorbed by wet skin. When arsenic enters the circulatory system at high amounts through percutaneous absorption, there is a risk of carcinogenesis [17]. It was advised that the cosmetic products comprise fewer than 5 ppm of metal contaminants based on recognized toxicity research. Arsenic, which is common in water, soil, and food, can quickly enter the human body when swallowed. The majority of dust parts are placed on the lining when the air containing arsenic dust is inhaled [18]. The material that goes through the skin into the body is very small, which limits internal exposure to arsenic; therefore, there is highly unlikely that it will lead to arsenic poisoning [19]. A simple diffusion mechanism enables the majority of arsenic to enter the body in the trivalent inorganic form As(III) [20]. Only a limited amount of pentavalent inorganic arsenic can move through cell membranes via an energy-dependent transport process prior to being converted into trivalent arsenic. As arsenic is excreted through urine, both organic and inorganic, most inorganic arsenic could be removed in a few days, although some will linger for months. Organic arsenic is normally removed faster than inorganic arsenic within a few days [21]. Arsenic and other metals in soil have a seriously detrimental effect on health in various places of the world. In Bangladesh and West Bengal, India, some of the worst occurrences of arsenic poisoning were observed where close to 43 million people drank arsenic-laden water [22]. WHO suggested the arsenic limit for water is 0.001 µg/L, although a level up to 0.05–3.20 µg/L has been found [23].

Epidemiological data show that environmental arsenic ranges from 7 to 18 µg/L in topsoils are certainly associated with the mortality and prevalence of AD [24]. In an epidemiological study with 434 human participants, it was found that low-level arsenic exposure was related to poor neuropsychological action [25]. Nevertheless, an additional study showed a positive relationship between cognitive ability and serum arsenic, which suggests that consumption of seafood arsenic (docosahexaenoic acid) plays an important role in delaying AD pathogenesis [26]. Another study has indicated that in rat cerebellar granule neurons, As exposure led to apoptosis and neurotoxicity via activation of JNK3 and p38 MAP kinase signaling pathways [27]. Importantly, animal and human studies have found that air pollution is also a source of exposure and can worsen the pathology of neurodegenerative disease, which may involve the development of neurotoxicity [28]. Thus, experimental, clinical, observational, and epidemiological studies have described that As causes AD pathogenesis. The relationship between environmental arsenic and incidence of AD is presented in Table 1.

## 3. Prevalence of Arsenic Exposure and Potential Risk of AD Development

While inorganic arsenic (As) is a well-known neurotoxic metalloid with adverse neurological and cognitive impacts, the consequences on the elderly have received less attention. Only a few investigations have looked at As exposure as a risk factor for developing Alzheimer’s disease [8]. According to a study conducted on rural and elderly people in Texas (Project Frontier), poor cognitive ability and memory (after being adapted to confounders such as ApoEε4) revealed early indicators of Alzheimer’s disease, which are associated with long-term exposure to low arsenic levels (3–15 µg/L As in water) [29]. Occupational arsenic exposure was associated with memory loss and cognition function in humans [30]. In Thailand, India, Bangladesh, Mexico, Taiwan, and mainland China, chronic arsenic exposure in the air or drinking water has consistently been found to be related to memory reduction as well as intellectual capabilities in adolescents or children [7]. It has been documented that there was a potential connection between arsenic exposure in drinking water and cognitive dysfunction among adults living in a rural area of Cochran County, Texas [25]. Furthermore, arsenic exposure in childhood might be linked with lower education levels and AD. Therefore, arsenic exposure appears to have a more potent effect on cognitive function in AD throughout the lifetime, consistent with the lifelong-exposure hypothesis for AD.

## 4. Molecular Basis of Arsenic Toxicity and Its Implication in AD Pathobiology

**Figure 2:**
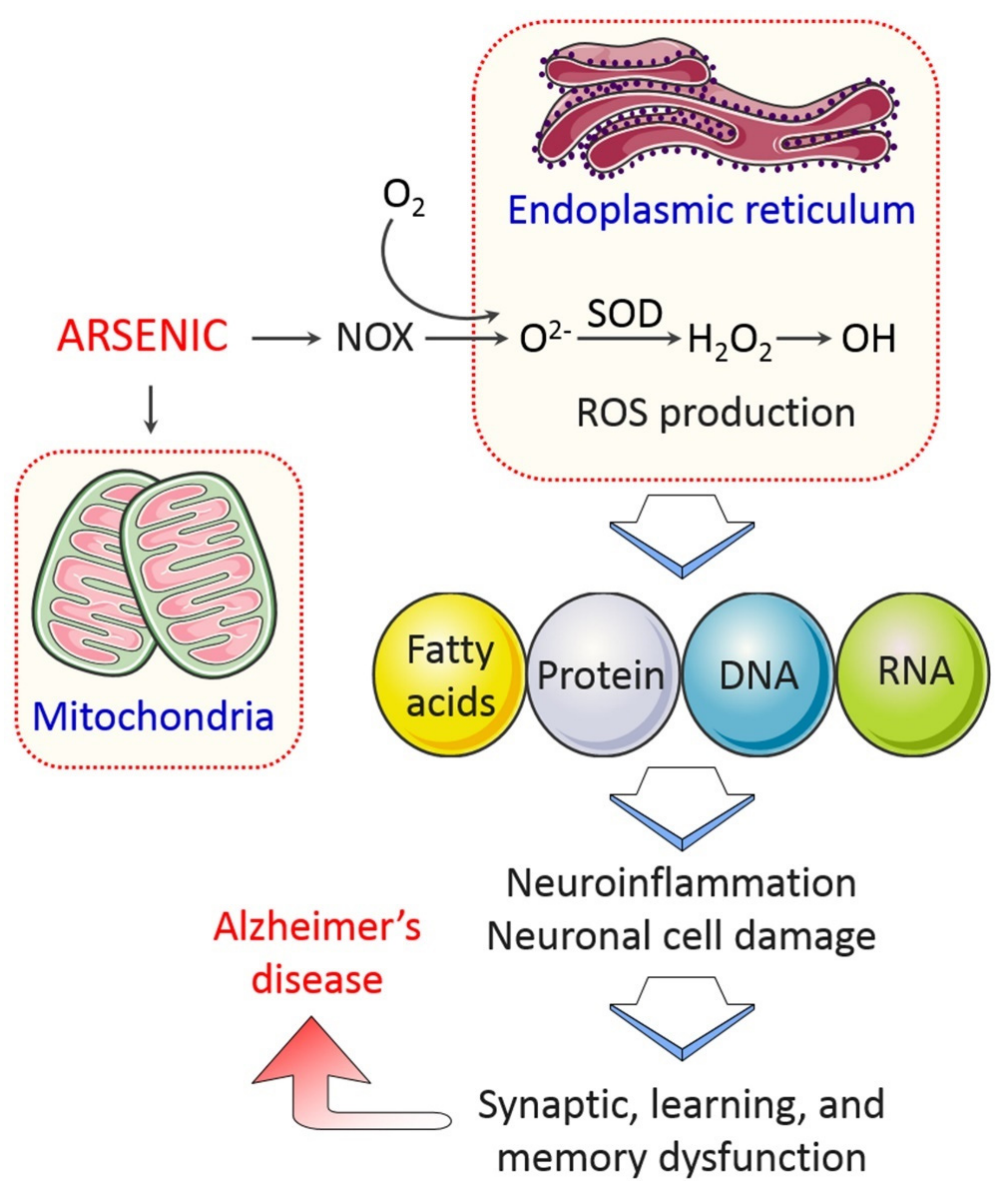
Arsenic-induced mitochondrial dysfunction and ROS generation in AD. As induces ROS production by mitochondrial dysfunction. NADPH oxidase (NOX) contributes to generate superoxide anions, which leads to the release of ROS in cells. ROS generation impairs fatty acids, proteins, and DNA, inducing neuroinflammation and subsequently causing cognitive dysfunction in AD.

Arsenic exposure has a direct impact on its toxic effects, but its exact molecular mechanism of action is not fully understood yet. There are several hypotheses: one is the generation of reactive free radicals, which oxidize cellular components such as lipids, proteins, and DNA, ultimately causing oxidative stress and subsequent damage to cells (Figure 2). Arsenic-induced oxidation of DNA leads to a reduction in the antioxidant capability of rodents’ brains and protein thiols in the hippocampus, striatum, and cortex, resulting in downregulating ATP-synthase and encouraging peroxidation of lipids in rat brains [31]. In drinking water, 0.005 to 0.02 ppm as well as 0.01 to 0.05 ppm environmental arsenic exposure in humans and mice, respectively, enhances oxidation of DNA and proteins, and oxidative damage with inflammatory responses [32]. All this evidence suggests that arsenic is a possible etiologic factor for the oxidative stress hypothesis in AD pathogenesis, which could anticipate that levels of oxidized metabolites of RNA, DNA, fatty acids, and proteins increase in AD brains [9,33,34].

**Figure 3:**
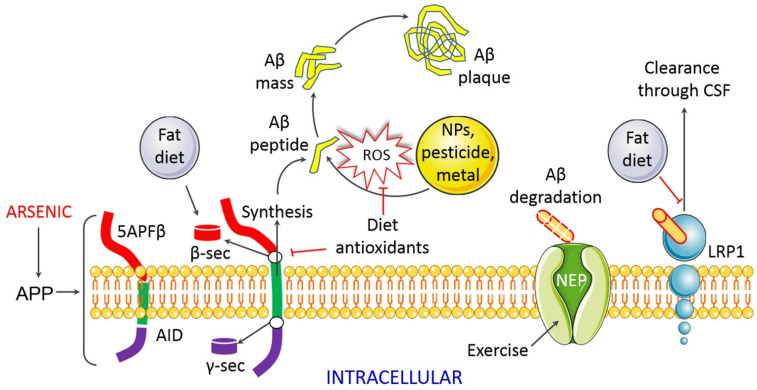
Arsenic induces Aβ accumulation during AD pathogenesis. APP cleaves through the enzymatic action of β- and γ-secretase, which subsequently produces Aβ plaque. Dietary antioxidants may hinder the formation of Aβ plaque and ROS, and thereby may prevent AD progression.

Arsenic exposure has been found to reduce memory as well as learning ability in animal models where offspring of mice treated with 0.75 mg/kg of arsenic during pregnancy displayed neurobehavioral retardation in fetal origin [35]. Inorganic As (20 mg/L) induced severe spatial memory losses in mice during pregnancy and early postnatal life [7]. The modification of the amyloid pathway is a plausible explanation of cognitive and memory problems due to As exposure [3,36]. It has been reported that incubation of cholinergic SN56.B5.G4 cells with organic dimethylarsinic acid (DMA) (5–10 μM/12–24 h) increased Aβ levels [9]. However, Tg2576 mouse neurons have been shown to have similar effects (a murine model that overexpresses a mutant form of APP most used in AD) [37]. The effects of DMA are presumed to be attributable to higher Aβ anabolism (greater APP explication), although the Aβ degradation process has not changed. The method by which As produces excess Aβ is not known, but the inflammatory response in the brain and the oxidative stress are associated, which is compatible with the hypothesis of Alzheimer’s pathobiology (Figure 3) [33,38].

### 4.1. Mechanism of Arsenic-Induced Neurotoxicity

Human exposure to As is related to extensive neurological problems, including poor concentration, impaired memory, Guillain–Barre-like neuropathy, encephalopathy, verbal comprehension, Parkinson’s disease, and peripheral neuropathy [34]. The exact mechanisms that underlie arsenic neurotoxicity are largely not yet known. However, experimental evidence suggests that pathological factors including oxidative stress, inflammation, mitochondrial dysfunctions, ER stress, apoptosis, and impaired protein homeostasis are supposed to be implicated in AD pathobiology [39].

#### 4.1.1. Oxidative Stress

Oxidative stress plays a critical role in the pathobiology of various neurological disorders, including AD [40]. Oxidative stress is accompanied by an increase in reactive oxygen species (ROS) and lipid peroxidation, reduced levels of superoxide dismutase and glutathione (GSH) [41], and constitute the most important mechanism hypothesized for arsenic-mediated neurotoxicity. Intracellular ROS production appears to be the key mechanism of arsenic-induced neurotoxicity. Arsenic-methyltransferase, possibly a protein-binding substrate in the brain, is involved in the processing of inorganic arsenic (iAs) in the presence of GSH, monomethylarsonic acid (MMA), and DMA [42]. Depletion of GSH, therefore, hampers the metabolic processing of IAs. Furthermore, oxidative damage has been associated with the rise in BACE1 activity and consequently with Aβ levels in animal brains exposed to iAs over time [3]. Oxidative stress thus seems to play an essential role in arsenic-mediated neurotoxicity.

#### 4.1.2. Neuroinflammation

Neuroinflammation is another crucial event that largely contributes to AD pathobiology [43]. Evidence suggests that arsenic plays a vital role in neuroinflammation via activation of microglia through secretion of pro-inflammatory cytokines, which could damage cognitive function, intellectual ability, and learning and memory functions [11,44]. Arsenic administration augmented ROS-mediated expression of pro-inflammatory cytokines in rats via activation of MAP kinase and protein kinase C through increased mRNA levels of pro-inflammatory markers such as TNFα, IL-1β, and IFNγ with protein expression of TNFα and IFNγ [45]. Intake of excess arsenic implicated MBP-activated autoimmunity and neuroinflammation in athymic nude mice with depleted T cell populations in peripheral lymphoid organs [46].

#### 4.1.3. Mitochondrial Dysfunction

**Figure 4:**
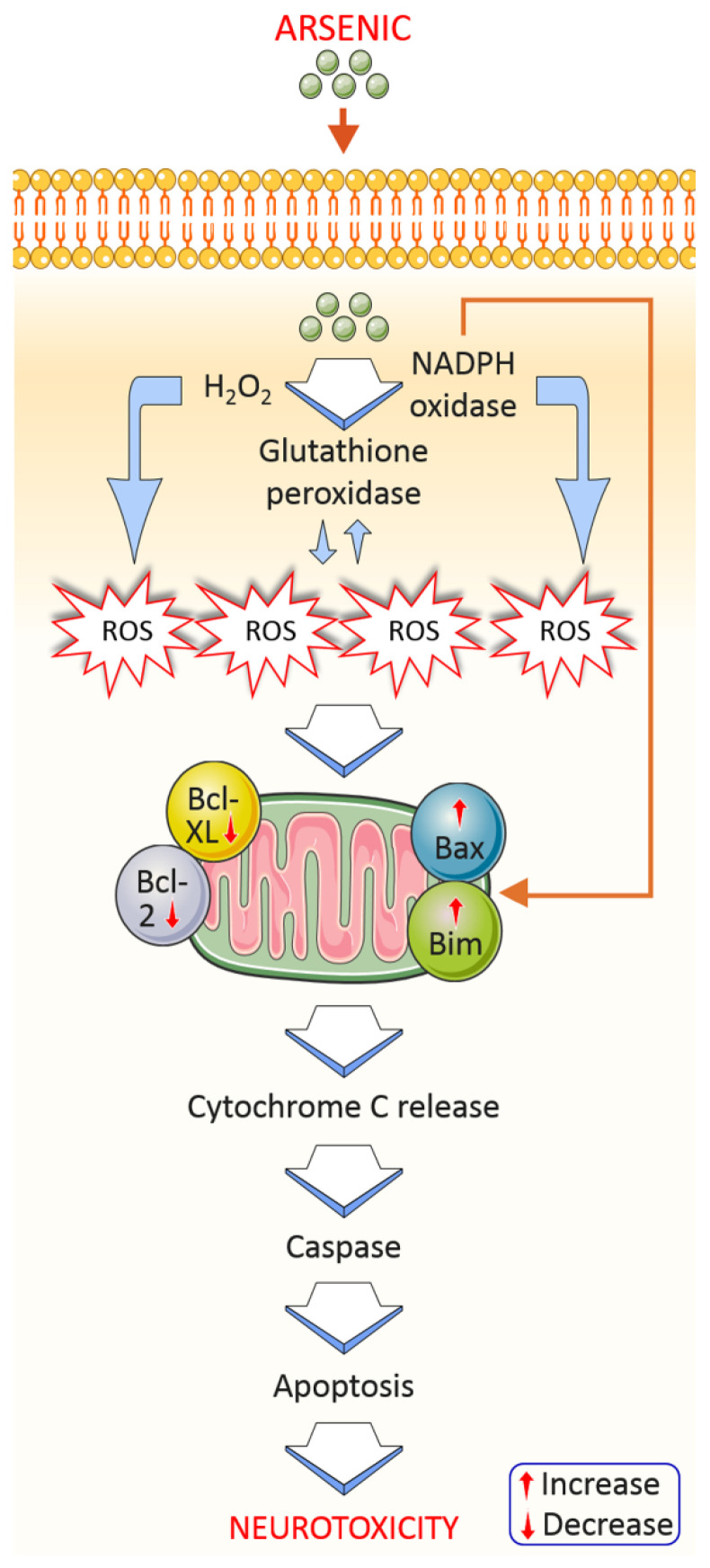
Arsenic exposure induces apoptotic factors from mitochondria to cause neurotoxicity. Mitochondria are the main target in arsenic-mediated neurotoxicity. Arsenic-mediated oxidative stress by hydrogen peroxide and NADPH oxidase generates ROS production, which can disrupt membrane potential resulting in cytochrome c release. Mitochondrial apoptotic markers, for example, Bax/Bim and Bcl-XL/Bcl-2, and numerous inflammatory markers (NF-kβ) alter and activate caspase activation, which causes cell damage and cell death via apoptosis.

Mitochondria occupies a central position in cellular bioenergetics. Mitochondrial dysfunction and oxidative stress are closely linked and, together with inflammation, constitute a pathological triad [47]. Understanding molecular mechanisms that underlie mitochondrial dysfunction might be useful to progress therapeutic approaches to be effective against arsenic-mediated neurotoxicity. Several reports have described impairment of brain mitochondria during arsenic-mediated toxicity [48,49]. Mitochondrial ROS accumulation has been found after arsenic treatment in rat brains [49]. It has been indicated that Aβ lowered the activity of mitochondrial complex I in As-induced 3xTgAD mice. This study indicates that amyloid aggregates can hinder mitochondrial function. In this hypothesis, reductions in ATP generation are responsible for the endoplasmic reticulum (ER) stress leading to the accumulation of the malfunctioning proteins in its lumen [50]. Arsenic has shown its mutagenic response via disruption of mitochondrial function. As-induced perturbation to mitochondrial oxidation caused production of excess superoxide anions, which when reacted with nitric oxide generate highly reactive peroxynitrites [44]. Arsenic toxicity affects the mitochondrial membrane potential via generating ROS and DNA fragmentation, and ROS overproduction is associated with apoptosis induction by the release of cytochrome c, which activates the caspase pathway (Figure 4) [51]. Thus, mitochondria are the major target in arsenic-mediated neurotoxicity and the subsequent neurological deficits in AD.

#### 4.1.4. Endoplasmic Reticulum (ER) Stress

Current evidence suggests a relationship between ER stress and arsenic-induced neurodegenerative diseases [3,52]. Arsenic has been found to induce endoplasmic reticulum (ER) stress via accumulation of misfolded proteins, which results in neurotoxicity and cell death [53], although the underlying molecular mechanisms are not well understood. Recently, it has been described that arsenic-mediated ER stress and neurotoxicity are associated with early neurodevelopment, which can be alleviated by microRNA-124 [52]. Additionally, in MIN6 cells, arsenic impaired mitochondrion function through diminishing mitochondrial membrane potential and decreased cytochrome c release, which cause mitochondrial ROS generation [54]. 

#### 4.1.5. Apoptosis

Arsenic toxicity involves apoptosis as a common phenomenon of cell death. Arsenic-induced neurotoxicity has been involved in apoptosis induction in the cerebral neurons via activation of JNK3 and p38 mitogen-activated protein kinase (p38MAPK) pathways [27]. Recently, it has been found that arsenic prompts neuronal apoptosis through upregulating Bax levels as well as decreasing Bcl-2 protein [55]. Additionally, arsenic-induced keratinocyte apoptosis in the extrinsic apoptotic pathway involves Fas/FasL, which is correlated with modifications of AP-1 and NF-κB pathways [56]. Arsenic-induced neuronal cell death involves activation of autophagy-dependent apoptosis through inactivation of the Akt pathway and activation of the AMPK pathway [57].

#### 4.1.6. Impaired Proteostasis

Protein quality control systems, also known as protein homeostasis or simply proteostasis, play a significant role in cellular physiological functions. Impairment in proteostasis leads to aberrant deposition of protein aggregates, which are characteristics of many neurodegenerative disorders such as Aβ aggregate in AD [58,59]. Compelling evidence from recent studies suggests that chronic exposure to inorganic arsenic can disrupt protein quality control and clearance systems, which contribute to the pathobiology of proteinopathic brain disorders such as AD. Arsenic-mediated post-translational modifications of proteins and disruptions of ubiquitination may culminate in impairment in proteostasis [60]. Genome-wide imaging screen analysis uncovers the molecular causes of arsenite-mediated protein aggregation and toxicity studied on *Saccharomyces cerevisiae* deletion mutants [61]. Recently, it has been found that three ER stress sensors viz. ATF6α, IRE1α, and PERK have an important role in ER stress-related autophagy, mitochondrial dysfunction, and unfolded protein response (UPR) in arsenic-induced malignancies to identify vital targets for therapeutics of neurodegenerative and cancer prevention [62]. Furthermore, arsenic prevents SNARE complex formation via increasing SNAP29 O-GlcNAcylation, which perturb proteostasis, and transfection of O-GlcNAcylation-defective CRISPR-mediated SNAP29 knockout cells eliminates arsenic-induced autophagy inhibition [63]. Additionally, arsenic has been found to induce autophagy inhibition and ER stress induction, which suggests low arsenic-mediated recovery of oxidative stress, and restoring proteostasis by the autophagy pathway [64]. Particularly, arsenic-mediated UPR activation is related to the accumulation of p62 and LC3 protein aggregates in the sequestration of autophagic protein clearance [65], and activation of UPR as well as formation of aggregated protein might be targeted to the lysosomal degradation of proteostasis.

#### 4.1.7. Impaired Calcium Signaling

**Figure 5:**
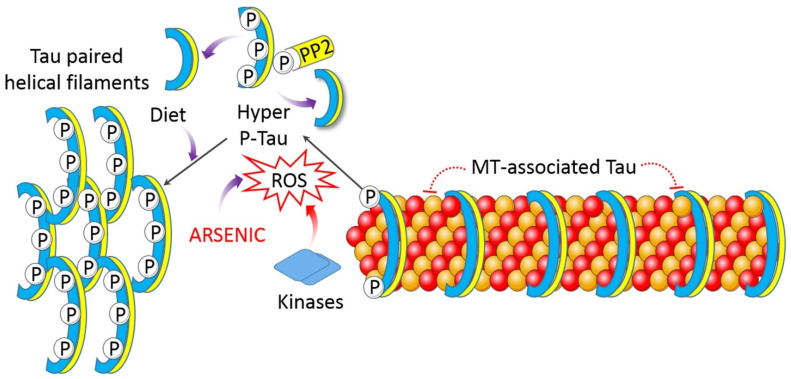
Arsenic influences tau hyperphosphorylation in AD. Hyperphosphorylated tau aggregates and acts on mitochondria, triggering energy deficit.

Arsenic-induced mitochondrial dysfunction causes a decline in ATP generation, which is responsible for ER stress, leading to calcium build-up in intracellular compartments and impairment in calcium signaling, most likely due to a lack of recovery systems [50]. Altered calcium signaling can cause cognitive impairment or the development of tau hyperphosphorylation by activating protein kinases such as GSK-3. Hyperphosphorylated tau and amyloid aggregates interact with mitochondria, generating complex I shortages and leading to an establishment of a vicious cycle of energy deprivation and proteostasis (Figure 5) [66].

## 5. Management and Control of Arsenic-Induced Neurological Deficits

Although the epidemiological reports and experimental evidence clearly demonstrate that high exposure to arsenic results in abnormalities in the developing brain and cognitive deficits in adults, the specific management strategy that can effectively address arsenic-induced neurological deficits is yet to be developed. However, the approaches such as administration of biological trace elements (zinc and selenium), antioxidants and arsenic chelators, high-protein diets, and exposure to an enriched environment could be the possible strategies that can ameliorate arsenic-induced systemic deficits.

**Figure 6:**
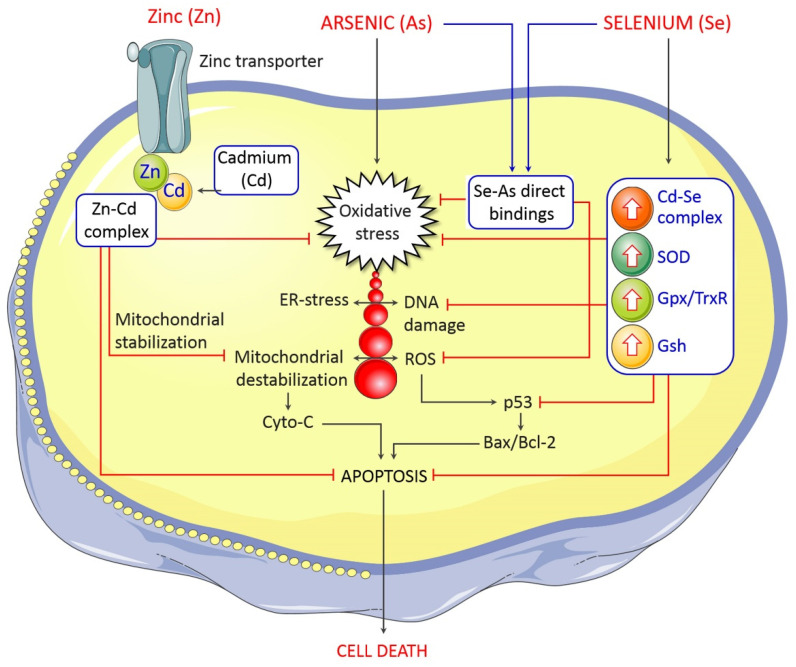
Possible protective mechanism of selenium and zinc against As toxicity. Arsenic induces oxidative stress, followed by DNA damage, ER stress, and mitochondrion dysfunction, which subsequently trigger apoptosis. Se–As complex inhibits oxidative stress, and Cd–Se prevents ROS production, DNA damage, and apoptosis. Additionally, Zn–Cd complex activates mitochondrial stabilization and inhibits oxidative stress and apoptosis.

The forefront strategy that has been shown to be effective in alleviating arsenic-induced toxic effects is the dietary inclusion of trace elements such as zinc and selenium. Zinc can prevent arsenic-induced neurotoxicity in fish models by preserving the blood–brain barrier and attenuating apoptosis and autophagy dysfunction [67]. In several other studies with common carp, zinc supplementation also has been shown to be effective in protecting against arsenic-induced toxicity in the heart [68], kidney [69,70], liver [71], spleen [72], and pancreas [73]. In a rat model of chronic arsenic toxicity, zinc supplementation can protect against damages to the liver and kidney [74]. In a neuronal cell line, zinc was shown to alleviate arsenic-induced apoptosis (Figure 6) [75]. Early-stage zinc supplementation in pregnant women can prevent preterm birth induced by arsenic toxicity, as observed in a rural Bangladesh birth cohort [76]. Another vital trace element with antioxidant potential is selenium that has been reported to be protective against arsenic toxicity. Selenium co-administration can protect against arsenic-induced behavioral deficits in rats through a mechanism involving anti-inflammation, antioxidation, and anti-apoptosis (Figure 6) [77]. In another study by Samad and colleagues, selenium supplementation through drinking water ameliorated arsenic-induced anxiety/depression and memory deficits in rats [78]. In chickens exposed to arsenic at a subacute dose, selenium (up to 10 mg/kg) prevented oxidative damage, neurotransmitter disorders, and apoptosis in the brain [79]. Additionally, selenium was reported to be effective in ameliorating arsenic-induced damage to other tissues including the liver [80], reproductive organs [81], and other organs [82].

In addition to biological trace elements, food-derived bioactive compounds that can attenuate oxidative stress and inflammation have protective potential against arsenic-induced tissue damage [83]. The most notable of the natural compounds are curcumin, quercetin, gallic acid, genistein, resveratrol, and thymoquinone, whose protective effects against arsenic toxicity have been supported by multiple studies in animals [84,85]. However, extrapolation of the beneficial effects of these compounds from preclinical evidence to clinical patients requires further validation. A limited number of studies of phytochemical effects on arsenic-induced toxicity in brain disorders have been reported. Green tea and vitamin C attenuated arsenic-induced lipid peroxidation in rat brains [86]. In addition, the leaf extracts of *Annona muricata* reduced arsenic-mediated neurotoxicity [87,88]. Besides, the treatment of arsenic intoxication is carried out mainly by chelation therapy using dimercaprol and aqueous garlic extract in A375 cells [89]. Garlic extract is a potential antidote to the toxic effects of sodium arsenite in mice [90]. Allicin, a bioactive compound of garlic, decreases arsenic-induced oxidative stress and toxicity in mice [91]. The aqueous extracts of garlic and its derivative, allicin, take part in chelation of arsenic [92]. Additionally, combined effects of various plant extracts have been recorded for arsenic-induced hematological, renal, and hepatic alteration in experimental animals [93,94,95]. Nanocapsulated quercetin prevented arsenic-induced damage in various organs, including brains of rats and mice [96,97]. 

Access to a high-protein diet also can help mitigate arsenic toxicity. Evidence suggests that ensuring an enriched environment can be a prospective strategy against arsenic-induced neurological problems, probably by alleviating depression and stress [7]. Moreover, conventional therapy that uses various chelating agents such as dimercaprol (BAL, British antilewisite), dimercaptosuccinic acid (DMSA), and penicillamine still may benefit arsenic-induced neurological deficits [98]. Beyond the management strategies outlined above, preventive measures such as access to arsenic-free drinking water, avoiding occupational exposure to arsenic, avoiding risk factors to neurological disorders, and provision of nutritious foods need to be considered for the ultimate prevention of As-induced health consequences, including neurological deficits [99].

## 6. Conclusions and Future Directions

Health consequences of As exposure represent one of the devastating setbacks of environmental pollution in human history. As reported in various epidemiological and experimental studies, arsenic extends its toxic effects to a number of vital organs, including the brain, where it causes neurodevelopmental abnormalities in childhood and cognitive deficits in adults. Although the underlying precise mechanisms of As-induced neurotoxicity have not yet been determined, the information from this review shows that the changes caused by arsenic exposure coincide with the pathological progression, clinical symptoms, and biochemical features of AD. While acute As toxicity can be managed by clinical use of specific antidotes, existing strategies to manage chronic As exposure are limited to supportive only. Extensive research involving animal models and appropriate human subjects is crucial to explore the detailed molecular mechanisms in order to design an effective therapeutic strategy against this menace.

## Figures and Tables

**Table 1:** Environmental arsenic factors highly related with AD pathogenesis.

Dose and Level of Arsenic	Study Model	Effects/Molecular Mechanism	References
13–15 mg/kg.	Mortality data by WHO, epidemiological and geological data.	Induce AD and other dementias as a composite morbi-mortality index.	[24]
Sodium arsenite (10 µM).	Cerebellar granule neurons of rats.	Activation of p38 and JNK3 MAP kinases cause cerebellar granule neurotoxicity and apoptosis.	[27]
Groundwater long exposure of 240.15 ± 182.96 µg/L.	Longitudinal epidemiological human study.	Low and long As exposure linked to global cognition function.	[25]
Drinking water (10 µg/L).	Rat and human brain.	Tau hyperphosphorylation and APP over transcription.	[28]

## Data Availability

Not applicable.
